# Working in dissonance: experiences of work instability in workers with common mental disorders

**DOI:** 10.1186/s12889-017-4388-3

**Published:** 2017-05-18

**Authors:** Louise Danielsson, Monica Bertilsson, Kristina Holmgren, Gunnel Hensing

**Affiliations:** Section for Epidemiology and Social Medicine, Department of Public Health and Community Medicine, Institute of Medicine, University of Gothenburg, Box 453, 405 30 Gothenburg, Sweden

**Keywords:** Depression, Anxiety, Work ability, Grounded theory, Qualitative research

## Abstract

**Background:**

Common mental disorders have a negative impact on work functioning, but less is known about the process when the functioning starts to destabilize. This study explores experiences of work instability in workers with common mental disorders.

**Methods:**

A grounded theory study using a theoretical sampling frame, individual in-depth interviews and a constant comparative analysis conducted by a multidisciplinary research team. The sample involved 27 workers with common mental disorders, currently working full or part time, or being on sick leave not more than 6 months. They were women and men of different ages, representing different occupations and illness severity.

**Results:**

A general process of work instability was conceptualized by the core category Working in dissonance: captured in a bubble inside the work stream. The workers described that their ordinary fluency at work was disturbed. They distanced themselves from other people at and outside work, which helped them to regain their flow but simultaneously made them feel isolated. Four categories described sub-processes of the dissonance: Working out of rhythm, Working in discomfort, Working disconnected and Working in a no man’s land.

**Conclusions:**

The experience of work instability in CMDs was conceptualized as “working in dissonance”, suggesting a multifaceted dissonance at work, characterized by a sense of being caught up, as if in a bubble. Focusing on how the worker can re-enter their flow at work when experiencing dissonance is a new approach to explore in occupational and clinical settings.

**Electronic supplementary material:**

The online version of this article (doi:10.1186/s12889-017-4388-3) contains supplementary material, which is available to authorized users.

## Background

The negative and often long-term impact of common mental disorders (CMDs) on work functioning and sickness absence, encompassing mild to moderate depression and anxiety disorders, is well documented [1–3]. It is particularly worrying among younger age groups, with high and increasing disability due to CMDs [4]. Not only is this an individual or organizational problem, but an alarming societal burden and public health issue. Mental disorders are a leading cause for disability worldwide, and depressive disorders are estimated to count for 40% of disability adjusted life years [5]. A conservative estimate put the costs of mental illness at 3-4% of the gross domestic product in the European Union [6]. Although most people with CMDs are in fact employed and working, the major cost of CMDs is due to reduced work performance and productivity [7, 8]. To reduce the burden of CMDs in the workforce, maintaining workers’ capacities and quality in their work roles are important goals for mental health promotion [9].

Thus, health policies need to focus on what can be done to support people with CMDs *while still* working, to prevent sickness absence, disability and premature exit from the labour market [6]. This calls for a deeper understanding of how the functioning at work changes in the presence of CMDs, in order to recognize when early negative fluctuation occurs and provide the necessary support. In particular, the process prior to sick leave needs exploring [10].

From a qualitative perspective, working while depressed and anxious can mean an unfamiliar experience of feeling less “at home” with one’s job tasks and environment, interpreted as being like a guest in one’s own working life [11]. The work experience entails exacerbated vulnerability in the challenge of recovering [12]. Workers with CMDs emphasize the need for support from their managers [13] but also feel reluctant to let managers and colleagues know about their problems [14].

Full-time workers with CMDs report similar levels of symptoms as experienced by those on partial sick leave [15]. Hence, the severity of the disorder does not seem to fully explain the level of work impairment. Also, both work-related and disease-related factors seem to predict recurrent absence in CMDs [16]. The complex phenomenon of functioning at work needs to be examined using frameworks that acknowledge the interplay between the person, the work tasks and the work environment [17, 18], and between working life and private life [19]. The psychosocial work environment affects the worker’s health [20] and this interaction is suggested to be reciprocal [21]. This means that the worker’s health will also affect the environment and the worker’s possibility to be influenced by the environment.

An innovative approach is to view the individual’s functioning at work in terms of a dynamic balance. *Work instability* is a relatively new concept, previously explored in long-term somatic conditions such as rheumatoid arthritis, traumatic brain injury and multiple sclerosis [22–24] and in specific occupational contexts such as that of manual workers [25, 26]. The term work instability was originally coined to describe the extent of “mismatch” between functional capacity and work demands at a given time, and its impact on job retention [22]. Thus, the concept encompasses the assumption that the vocational impact relates to the interaction between the individual and their work demands. Here, we understand “work demands” as involving both tasks and environment. Work instability is currently defined as “a state in which the consequences of a mismatch between an individual’s functional and/or cognitive abilities and demands of their job can threaten continuing employment if not resolved” [22]. In this state, the worker is vulnerable to sick leave, but also to potential future disability and job loss. Identifying the presence and extent of work instability can help timing vocational support or other proactive interventions [22].

However, in order to do so, one must know what to look for. Work instability in CMDs has not yet been explored. Since CMD symptoms often affect the person for a long period of time, it is fair to assume that working while depressed and anxious entails a process of more, or less, instability. A deeper understanding of how the functioning at work destabilizes and what this experience means to the workers may contribute to new insights to support mental health at work and inspire interventions. Thus, the aim of this study was to explore experiences of work instability in workers with CMDs.

## Methods

This study used grounded theory, which departs from symbolic interactionism, pragmatism and social constructivism [27–29]. These philosophical roots of grounded theory share two principles that were important for our choice of method. Firstly, phenomena are conceived as dynamic. Secondly, the research, grounded in empirical data, seeks not only to uncover processes per se, but also to reveal how people respond to change in these processes [29]. We used the Consolidated Criteria for Reporting Qualitative Research (COREQ) checklist [30] while reporting the study.

The research team consisted of four researchers with professional backgrounds in physiotherapy, occupational therapy and social work, all with previous experience of qualitative research. Theoretically, the pre-understanding at hand was primarily influenced by the person-environment-occupational model [17] of work functioning outlined in the background, and by a phenomenological perspective on depression and anxiety, characterized by a sense of entrapment and disruption of the person’s interaction with the world [31].

### Participants

We included employed adults with a current diagnosis of unipolar depression or anxiety disorder (codes F32-39, F41 and F43 in the ICD-10 Classification of Mental and Behavioural Disorders [32]). To cover the whole spectrum of CMDs, including depressive and anxious symptoms without a confirmed diagnosis [33], we recruited some participants on the basis of low perceived mental wellbeing. This was defined as scoring below 50 in the WHO-5 Mental Wellbeing Index [34], a brief scale with sound psychometric qualities assessing mood, energy, calmness, sleep and sense of purpose. The maximum score is 100, with scores below 50 indicating low mental wellbeing [34, 35]. The scale was used for all participants to measure current mental wellbeing. Among the whole sample, the WHO-5 score ranged between 16 and 84, median 32. Another criterion for inclusion was that all participants were working full-time or part-time, or were on full sick leave but for no more than 6 months [22, 23]. This criterion was meant to capture experiences at different phases of instability. Criteria for exclusion were diagnosed psychotic symptoms, substance abuse, and neuropsychiatric disorder.

To achieve a purposive sample, we created a theoretical sampling scheme regarding occupations, clinical status, age and sex. From December 2015 to June 2016, 30 participants were recruited through clinical collaborators in primary care (*n* = 19), as well as through a patient organization (*n* = 7) and public lectures (*n* = 4). The participants recruited in primary care were approached face-to-face by clinical collaborators with information about the study. They could choose whether they themselves wanted to contact the researcher (LD), or if they preferred the researcher to contact them by telephone. The other participants contacted the researcher by e-mail or telephone. All participants spoke on the telephone with the researcher before the interview. They were informed about the aim and procedure of the study and the researcher’s background through oral and written information. For different reasons, three of the recruited persons did not take part in the study. One person was excluded because the job contract expired, one person declined participation because of stress and one person did not respond to the researcher’s attempt to book the interview. The final sample consisted of 27 participants (see Table 1).

**Table 1:** Characteristics of the participants

		Number of participants Total sample *n* = 27
Age	19–30 years	10
	31–45 years	9
	46–66 years	8
Sex	Women	19
	Men	8
Marital status	Single	12
	Married/cohabitant	15
Children living at home	Yes	6
	No	21
Diagnosis	F32-33 Depression	9
	F41, 43 Anxiety disorder	13
	Undiagnosed / Low mental wellbeing ^a^	5
Specific job strains ^b^	Physical tasks (major part of work involves physical strain)	9
	Interpersonal tasks (major part of work involves relating to others)	14
	None reported	4
Job classification according to major groups of International Standard Classification of Occupations ^c^	Managers	2
	Professionals	12
	Technicians and associate professionals	3
	Clerical support workers	3
	Services and sales workers	5
	Plant and machine operators and assemblers	1
	Elementary occupations	1
Employment sector	Public sector	15
	Private sector	10
	Own company ^d^	4

^a^defined as scoring below 50 on the WHO5 Mental wellbeing index

^b^self-reported, answering yes/no to the question: Does a significant part of your work consist of...

^c^occupations among the participants were: child care worker, community health care worker, midwife, medical secretary, care auxiliary, medical laboratory scientist, engineer, cook, waiter, mechanical assembler, janitor, contact centre information clerk, artist, designer, actor, primary school teacher, secondary school teacher, professor, social worker, business controller assistant, business analyst, social welfare manager, project leader, audio technician

^d^Two participants were both employed and had their own companies

### Data collection

Individual interviews, one per participant, were conducted at a location convenient to each participant: a primary care centre, a public library or the participant’s home. Present at the interview were the participant and the interviewer. The first author (LD) conducted the majority of the interviews (*n* = 22). She is a female physiotherapist and PhD experienced in qualitative interviews and has worked in psychiatry and primary care, treating patients with mental health problems for 12 years. Another female physiotherapist, also with long (>20 years’) experience of treating patients with mental health problems, conducted a subsample of the interviews (*n* = 5) as part of her Master’s thesis.

The interviews used a thematic guide (see Additional file 1), starting with an open question: *Can you describe what an ordinary day at work is like for you?* The participant’s narrative set the course of the interview, with the interviewer using follow-up questions to elaborate on the participant’s descriptions. Probes were used to encourage participants to elaborate on: what it is or was like to work, adjustments they had to make, interactions at work, the work environment, their lifestyle, bodily experiences, and life outside work. In accordance with grounded theory, the interview was not static in its structure and content, but guided by a parallel comparative analysis, so that reflections on previous data would generate new topics for discussion [27, 29, 36].

The interviews lasted between 23 and 96 min, the median duration was 48 min. They were digitally audio-recorded and transcribed verbatim. Additional to the textual data produced from the transcript, data also contained the interviewer’s notes and memos connected to the interviews and three pages of sketches produced by one of the participants. The participants were encouraged to contact the interviewer afterwards with questions or comments, and two of them did. In both cases, it was to add an experience to the interview, which was added as a note to the data.

### Data analysis

In the constant comparative analysis [27–29], the researchers’ reflections and notes enriched the continuously collected empirical data. The first author (LD), who performed most of the analysis, took notes during and after the interviews regarding themes, new aspects and particular expressions used. The transcribed interviews were analysed line by line in an open coding process using NVivo 11 for Windows (QSR International Pty Ltd., Victoria, Australia). Stepwise, a subsample of interviews (*n* = 15) was independently coded by the three co-authors. Thereafter, all authors reflected on the data in joint discussions to provide input to the analysis. Thematic content was reflected on and themes that needed more probing were highlighted. For example, one key theme that arose from these reflections on the data was the participants’ experience of a bubble/shelter/shield. In the subsequent interviews, the theme was elaborated on with the participants: What does this experience mean to you? What do you do when it occurs?

**Fig. 1:**
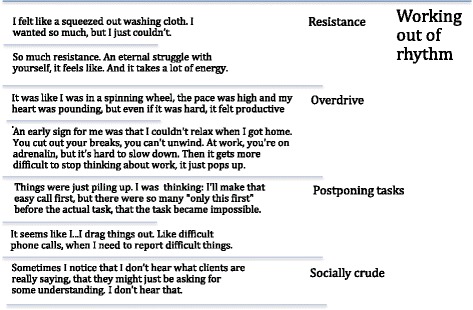
Example of the coding process grounded in empirical data

**Fig. 2:**
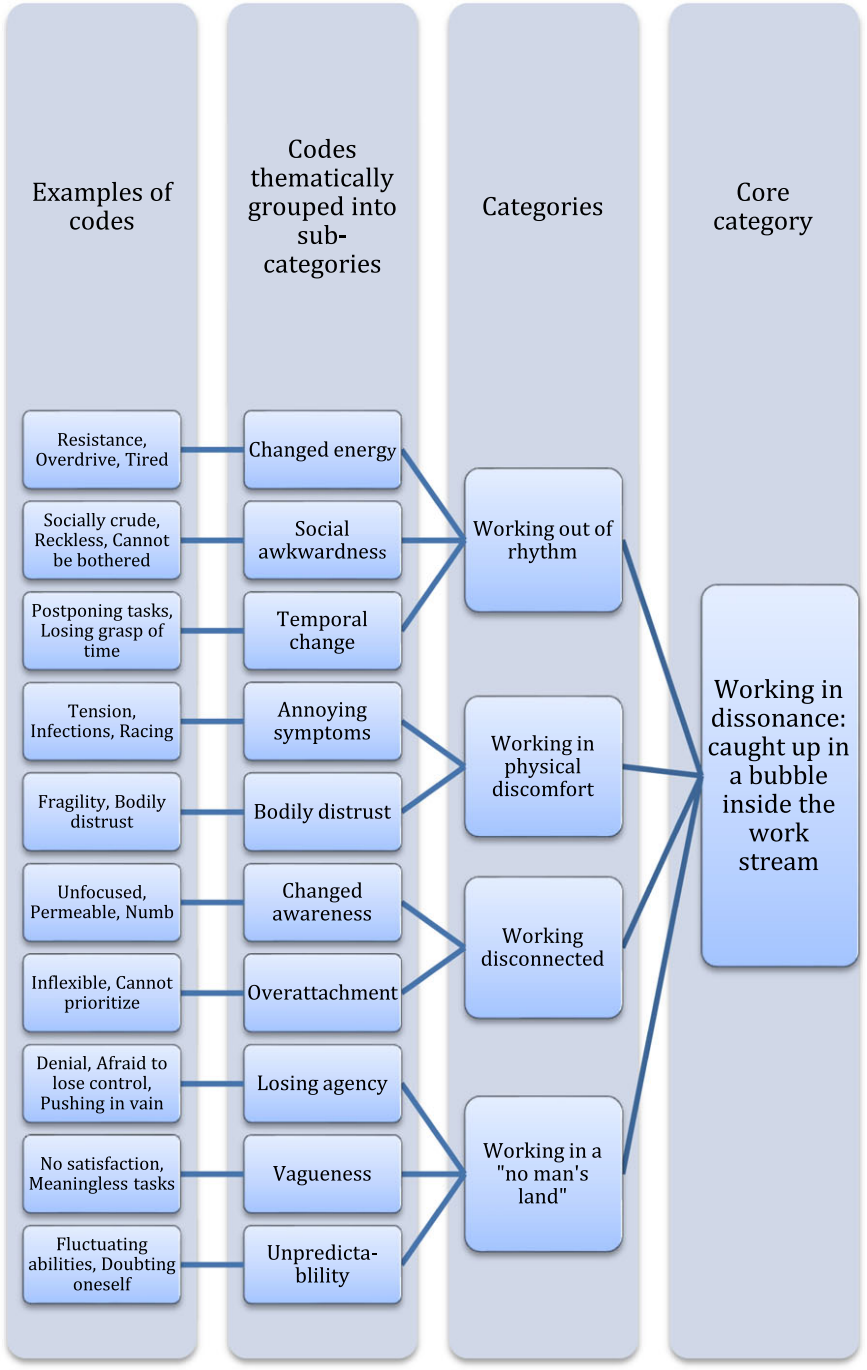
Schematic presentation of subcategories and categories

Codes from the open coding process were compared, including the identification of divergent cases, and grouped to create subcategories, see Fig. 1 for example. Subcategories were developed into categories, higher in level of abstraction, describing the “cornerstones” [29] of the general pattern of work instability in CMDs. The subcategories are presented in Fig. 2, showing the abstraction into categories. In the selective coding, the categories were then unified around a core category, to explain the central process identified in the data. Saturation was reached when the final interviews did not contradict the patterns of the interpretation, though providing more nuance. The description and headings of categories were elaborated in a writing–rewriting process [27].

The first author (LD) reflected on the results with clinical collaborators, with patients who attended a primary care clinic where she worked, and with the participants for member validation [37]. All participants were invited to give their comments on a summary of the results, which eight of them did. Overall, these comments were very brief but confirmed validity and recognition of the results. One example of a participant’s response was: *“Interesting to read, I can relate to most of it. Different individuals can surely experience these things differently, of course. I agree with the description that you want to withdraw from your colleagues, but sometimes withdrawing just isn’t possible and then you just have to endure.”*

We also discussed the results in two seminars with external researchers representing different professional backgrounds from the medical, behavioural and social sciences.

## Results

**Fig. 3:**
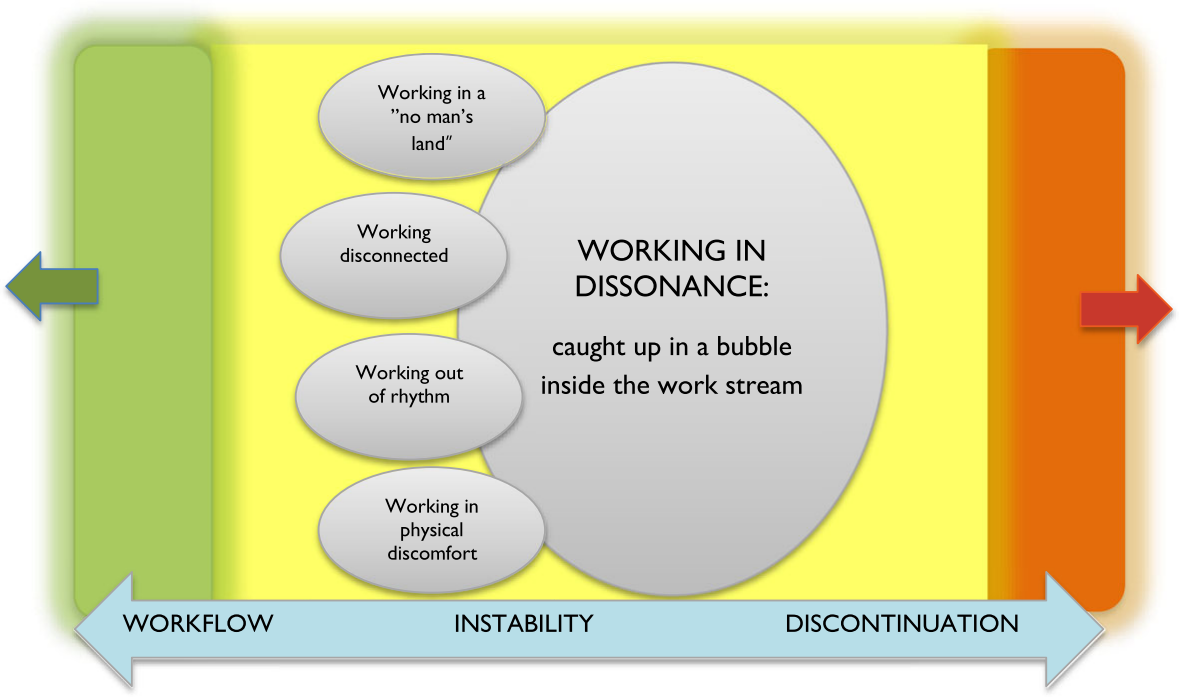
A visualization of the core process and the four sub-processes that make the core process likely to occur. The core category is understood as a process between workflow (*green*) and discontinuation (*orange*) of work, illustrating that the instability process can move in either direction: toward the worker re-entering flow or toward a state where work is no longer possible

A core category was formed that describes the general process of work instability, titled *Working in dissonance: caught up in a bubble inside the work stream*. The core category was understood as a process in which the ordinary flow of work is destabilized. The instability could be re-balanced to regain flow, or increase in the direction of work discontinuation, visualized by the horizontal continuum in Fig. 3.

Four categories were understood as sub-processes that make the core process likely to occur, see Fig. 3. They connect to the core process through the approaching experience of destabilization: that things do not run smoothly. While the core process represent the general pattern of work instability as a social process, the four categories illuminate different sub-processes: a temporal-spatial sub-process (*Working out of rhythm*), a physical sub-process (*Working in physical discomfort*), a psychological sub-process (*Working disconnected*) and an existential sub-process (*Working in a no man’s land*).

### Working in dissonance: caught up in a bubble inside the work stream

Work instability was understood as a dissonance in the otherwise smooth workflow, with which the participants struggled, involving work tasks, the work environment and other people. Working felt “out of sync” rather than feeling like a natural, everyday activity. The dissonance was experienced differently by different participants: to some it felt like an awkwardness that can be corrected; others had the sensation of a troubling distraction that is difficult to ward off, or a sense of complete derailing. The dissonance disturbed their ordinarily smooth workflow and made them feel apart from others. They worked caught up in a bubble, a shielded area, cut off and distanced from others. In one participant’s sketches, this was illustrated as a protective, surrounding bubble, which enabled work but created a sharp demarcation to others. This bubble provided protection, as it kept others at a distance. This enabled the participants to keep working by recapturing the focus and working at the required pace. In particular, this impacted on social interactions at work. The participants, both those with depression and those with anxiety, described an urge to shield themselves from noisy environments and other people at work. In sketches it was shown how imposed social interaction weakened the participant’s drawn bubble and increased stress. The participants urged to turn away from the common work stream. In doing so, they restored the flow but simultaneously felt detached.

Not only did the dissonance involve an urge to withdraw, it also meant being out of tune with other people at work. This experience strongly impacted work for those whose work entailed interpersonal tasks, which they usually enjoyed and were good at. For example, they described being annoyed by patients, impatient with students and inattentive to clients’ needs. These participants described difficulties to “tune in” to others at work.

The dual experience of the bubble as both protecting and providing detachment was present also in work adjustments. Although a private office space or a reduction in tasks could be facilitating, getting special treatment increased the sense of seclusion. Understanding and support from managers and colleagues were important, but the participants emphasized their professional role and were cagey about others recognizing, and about themselves admitting, presence of illness. Adding to the complexity of the dissonance were the participants’ descriptions of losing a sense of agency in the course of events: they recalled little awareness of *how* they had lost stability. In particular, this unseen instability appeared in the narratives about first-time episodes of CMD, They had just found themselves caught up in it; it was just there as a relentless process of its own.

#### Working out of rhythm

The participants described feeling out of time with their ordinary work rhythm, such as executing their tasks adequately and timely. The participants described that they needed to put in a lot more effort to perform. They felt slowed down when their effort seemed to be blocked and they were not moving forward. They struggled to regain their rhythm and catch up, for example by completing unfinished tasks at home, switching to a calmer room or scheduling less pressured routine tasks.

Working out of rhythm could also mean the participants’ experience of spinning faster and being sharper than their peers. Colleagues seemed slow, sloppy or insufficiently committed. The participants who described this problem said it was difficult to stop and switch off outside of work. They continued being in overdrive at home, doing frenetic housework, having a busy social life or performing intensive exercise. The overdrive experience was appealing as it gave a sense of high efficiency, but at the same time it was also unsettling:

So I keep a high pace and then when you work with someone who doesn’t keep that pace or doesn’t work really long hours, I can’t help thinking: “Why don’t they just work *more*?” (interview 26)

A salient finding in the interviews was that energy faded both because the participants were pushing themselves and because they were constantly in overdrive. In response, the participants started saving all their energy for work, reducing activities and social life outside work. One participant described herself as a zombie who woke up in the locker room at work, forced herself to get through the workday and then re-entered her zombie-like state when changing clothes to go home. In this way, the relationship between work and leisure time changed in favour of getting the work tasks done.

In this way, we interpreted this category, characterized by changed rhythms of time, space and energy, as a temporal-spatial sub-process that makes work dissonance likely to occur.

#### Working in physical discomfort

Salient in the data were the participants’ descriptions of a range of physical discomfort that interfered with their normal functioning at work. The discomfort did not always interfere with the work outcome, but caused an awkward feeling of the body being an obstacle to working. The participants described feeling estranged, tense, exhausted and weakened. They described muscle soreness as was usually associated with intense exercise, but in their case this was not the result of exercise. One participant felt that his body was fragile like a piece of paper. The participants described frequent infections and colds, resulting in a few days’ sick leave here and there. Normally not aware of their body at work, they now noticed that their physical discomfort hindered them at work:

My body is just supposed to be there for me, not messing things up. (interview 10)

The uneasy body could signal negative change and the need to take time out to recuperate. The discomfort could also serve a purpose, as one participant explained, saying that the high tension was what kept her going. For others, the annoying symptoms were more separated from the lived context. They were blamed on the body as a “thing” and as the frustrating reason for reduced abilities. For some, the body put a final stop to working:

I broke down at work. It was the end of a work day and I was going to a client and I just, I’m so tired, I just can’t anymore. So I can’t, I can’t move an inch, I can’t lift my legs, I just want to lay myself down here and now. (interview 3)

This category was characterized by bodily discomfort, the body calling for attention, which we interpreted as a physical sub-process that makes work dissonance likely to occur.

### Working disconnected

The participants described feeling disconnected both as an altered awareness and as a divide between how they felt and how they viewed themselves as workers. While feeling disconnected, they could get overly attached to assignments, unable to let go of details and responsibility. For workers with the possibility to check their job e-mails at home, this over-attachment was literally. The constant electronic link to work was hard for them to cut loose from. So they were on constant alertness regarding their work.

Keeping focused was difficult, in particular when the task required a more complex overview. It became tiring to follow a conversation and the participants needed to have information repeated to them. The altered awareness also included numbness and poorer coordination. They felt unsteady, stumbled and were butter-fingered, as for example reported by a teacher who was unable to complete scrapbook artwork with her pupils.

The disconnection was also experienced as the dissolution of one’s “filter” towards the working environment. This sometimes made it difficult for the participants to distinguish their own feelings from colleagues’ or clients’ discontent. They absorbed others’ negative feelings, which accentuated their own distress. For example, one participant working at a nursery described feeling overwhelmed by an upset parent, and unable to stop dwelling on the dispute. The opposite was also described: a numb detachment at work, as if “entering a phone booth”.

The participants gave rich examples of feeling disconnected in terms of “not being quite oneself” at work. Their view of themselves as efficient and reliable did not seem to fit their present experience. They described that they were pretending to be competent, putting on a façade. This gap between their “work self” and their private self could be distancing, protecting from difficult feelings. Alternatively, it was also expressed as a breathing space, in particular for those who also experienced strain in their private life. To take a break from distress at home and “dress” as a competent professional gave a sense of temporary relief and self-worth:

The days when I manage to hold that façade, it feels nice to be the professional, to be proud that I can actually manage it. (interview 4)

We interpreted this category, characterized by disconnection from one’s sense of presence, as a psychological sub-process that makes work dissonance likely to occur.

### Working in a “no man’s land”

The participants described that both their own work and the work environment no longer fully *belonged* to them. The ordinary sense of meaning and agency in their own working life was wobbling, making them feel lost. Unstable organizational conditions and vague tasks added to this challenge. The participants felt that their room for manoeuvre was shrinking and that this change was” just happening”, as a process that occurred by itself. To explain the feeling, one participant used the analogy of experienced time: you never really see how the hands of the clock move, but suddenly the hour has struck:

You just know that it’s moving, but you don’t see it. It changes all the time, you can’t put your finger on when it changes. And it’s the same with this, in a way, the longer it goes … That’s why you don’t notice it. (interview 27)

The unpredictable element was also described in terms of not being able to rely on a previously successful work adjustment to last. For participants who had gone through several episodes of illness, the unpredictability in itself became something they got used to, and thus became less stressful. The participants struggled to keep control, although their descriptions contained several metaphors that referred to something external calling the shots, like “an unstoppable train” or a “ship going down”, threatening their freedom of action:

Sometimes I feel like I’m walking in a very close corridor. And around it is this very unstable house of cards./…/. And this house of cards is so fragile, it must be protected. Because I’m so afraid that if it starts to crumble at one end, everything will fall apart! (interview 17)

The participants had a sense of working in a “no man’s land”, a surreal zone of feeling stuck between wanting to perform better and not being able to do anything, caught up in a self-repeating pattern. The experience, though coloured by their anxious or depressive state, had particular impact here as it reduced the work to monotony without progress. Achievements were cognitively noticed, but not felt, so they did not *mean* anything:

There are no goals to reach – the goal is only to continue. There is no joy in completing anything, because there is nothing at the other end. (interview 27)

In this way, these experiences of working in a “no man’s land”, related to the sense of meaning and coherence at work, was interpreted as an existential sub-process that makes work dissonance likely to occur.

## Discussion

To the best of our knowledge, this is the first study to explore experiences of instability in the work functioning among depressed and anxious workers. This is not to say that we saw the illness and the work instability as completely separate processes. Rather, we searched for patterns describing the “mismatch” [22] and destabilized work functioning from the workers’ perspective. The core category describes the general pattern of “what [was] going on” [27] in this process. There was disturbance in the ordinary fluency at work, linked to the workers distancing themselves from work in order to keep going. Four sub-processes illustrate different experiences of change that can lead to work dissonance, regarding time and energy, bodily sensations, sense of presence and meaning.

The core category *Working in dissonance: caught up in a bubble inside the work stream* sheds light on the finding that the worker’s distance towards tasks, the work environment and other people at work is double-edged. The tendency to withdraw as an illness experience has previously been reported [38], which manifests as a distance to work [13] and losing the feeling of being at home with oneself [39]. In this context, the bubble metaphor carries particular meaning, as the bubble seems to both hold back and enable work. We found that this experience was often described in the stories of depressed and anxious workers, and was shared by men and women with different family status and occupations, and those on sick leave as well as those fully working. This suggests that the core category captures something work-related, which is different from symptomatology. In an earlier study from our group, in which we examined experiences of capacity to work while depressed and anxious, we also found a distinction between the illness and the work capacity [11]. Findings in the previous study included difficulties adjusting to the surrounding workflow, pacing issues, inability to refuel, physical sensations, unfamiliarity and challenging social interactions, which corroborate our findings here. This study’s findings of the double meaning of “bubble” and the way it impacts the worker’s interaction with work, are new, as are the workers’ inability to “see” the process leading to instability.

Our core process can be discussed in relation to the concept of flow [40]. When in flow, the person performs at full capacity, in a state of equilibrium: a delicate balance between perceived capacities and opportunities. The balance is moderated by the perceived importance of the activity and by the achievement motive. However, flow can also be achieved at less demanding or important activities [41]. This is in line with our findings where participants described a sense of flow in less challenging routine tasks, even though these activities were not particularly fulfilling or meaningful. This finding is promising for future efforts to support workers to regain stability.

A disrupted being-with-the-world has been described in phenomenological studies on depression and anxiety, emphasizing changes in the embodied person’s interaction with the world [31]. This perspective was an important source of inspiration during the latter part of the analysis, likely to have influenced our conceptualization of work dissonance. In particular, our findings of temporal and interpersonal disturbances, such as the experienced slowness or overdrive compared to others at work and the difficulties to attune to others, connect to a recently proposed desynchronization [42] of time, space and inter-subjectivity [43].

We obtained rich data on the social domains of work, with the need for constant adaptation to other persons at work, be it managers, colleagues or clients. The ordinary day is full of encounters that put the person with CMDs under pressure. This may be a reason for adopting the shielded position as so many of the participants described. Some did not voluntarily adopt this position, but were pushed into it by their progressing illness. In line with previous research pointing to reduced biological flexibility in terms of heart rate variability [44] and gait patterns [45], we found that some participants presented a “social inflexibility” at work, for example the urge to avoid social interaction and the difficulty to let go of responsibilities*.* In several ways, the inter-subjectivity at work appeared to be very important in the work instability process.

The participants differed with regard to anticipated shame [46] about working differently because of CMDs. Most participants claimed performing adequately in the eyes of others, but felt bad for not living up to their own self-image and work ethics. This is in line with previous research [11, 13, 47, 48]. In a similar way, a strong individualistic view on work was discussed in a recent study on young workers, in which good work capacity was basically experienced as being a personal responsibility [49].

The results give insight into how the workers experienced, viewed and responded to the perceived changes in their functioning at work. Moreover, the analysis provides content to develop and enrichen the construct of work instability in CMDs. We found some aspects that are slightly different from the results in previous work instability studies. First, the work instability, in terms of the experience of dissonance and disturbed workflow, was described like a process rather than a state. Second, the workers in our study emphasized that their work experience was closely related to their life outside work, including stressors and recreation. Third, work and mental health were intrinsically linked for the workers: not only can CMDs influence work, but job stressors can hamper or facilitate recovery, and in some cases trigger the onset of CMDs [20, 50]. Elaborating on the previous definition (p. 4), we suggest that work instability in CMDs entails: “a process of experienced dissonance, where the consequences of mismatch between the worker’s abilities and demands of their job (involving tasks, social relations and environment), can threaten continuing work if not resolved”. Compared to the concept of work ability, the construct of work instability might introduce a more proactive approach in the prevention of impairment and sickness absence due to CMDs. Knowing more about the complex experience of dissonance presented in this study, involving the recognition of progressing signs of instability, can help depressed and anxious workers and their managers to understand changes at work. This understanding can guide the need for vocational adjustments, managerial and collegial support and external support such as health care providers, prior to sick leave.

### Strengths and limitations

Strengths of this study were the sampling scheme, the extensive data from in-depth interviews, the experienced and multidisciplinary research team and the validation efforts regarding both patients and external researchers [30, 37]. The recruitment strategy, of approaching both clinical and community arenas to recruit a purposive sample, ensured transferability of the results. It can be argued that the exclusion of substance abuse disorder limits transferability. Our reason for this exclusion was that we assumed that these workers would represent a particular subgroup, likely to describe their problems at work related to their addiction rather than to their CMD symptoms. In retrospect, since substance abuse is common in CMDs and potentially impacts on how the worker manages distress at work, this aspect warrants caution and provides a subject for further investigation.

Including some participants without a confirmed diagnosis may be regarded a limitation. We argue that it was essential to include them, to capture experiences that were less coloured by a medical diagnosis [33]. Also, this group of workers are of high interest, as they are at risk for future impairment and may benefit the most from preventive measures [51]. The use of the WHO-5 Mental Wellbeing Index helped to define low mental wellbeing in these participants.

The interviewers’ physiotherapy background and previous life-world led research may have influenced the follow-up questions in the interviews. The collaboratively designed interview guide and the continuous discussions parallel to data collection among all co-authors aimed to reduce bias.

The authors’ previous phenomenological work enhanced sensitivity toward making meaning, beyond the taken-for-granted, of the participants’ embodied experiences and actions. It also contributed to a stance of reflexivity among the researchers, emphasized in constructivist grounded theory [52, 53]. However, we saw a need to reflect on the methodological differences and the risk of method “slurring” [54]. The senior researchers in the group, who had conducted several grounded theory studies, provided other perspectives rooted in sociology and social constructivism, to the continuous analytic discussion.

Our study represents the worker’s perspective. To fully understand work instability in CMDs, other stakeholders such as policy makers, managers, colleagues and health care professionals would add important information [28]. Other sources of data, such as observations, could capture work instability in action. Possibly, this would add richer data about the “just-happening” dimension that we found in the workers’ accounts, that was difficult to elaborate on in the interviews. What exactly does this dimension mean – a blurred self-awareness? Not wanting to see or be caught up in something outside of oneself? If instability “just happens”, it is likely difficult for the workers themselves to notice instability at an early stage. Since this group of employees are at risk for recurrent absence [55], helping them to understand work instability at an early stage can be beneficial.

### Implications

CMDs in the workforce challenge welfare solutions and work places, and raising awareness of the complexity of work functioning is essential to develop new ways to prevent absence from work [6, 51]. Our findings can increase awareness among workers, employers, clinicians and policy makers, of how CMDs impact on work functioning. The workers’ awareness can be particularly relevant, considering how the results point to their difficulties to grasp what they are caught up in at the time. Possibly the results will encourage a shift of focus from the dichotomy of work ability/disability, to the more process-oriented view of managing work instability in the workforce.

## Conclusions

The experience of work instability in CMDs was conceptualized as “working in dissonance”, suggesting a multifaceted dissonance at work, characterized by a sense of being caught up, as if in a bubble. Understanding the duality of this bubble - both sheltering and entrapping – can be helpful to support workers in a sensitive way: to allow space but be attentive to signs of isolation. Focusing on how the worker can re-enter their workflow and sense of participation when experiencing dissonance is a new approach to explore in occupational and clinical settings.

## Additional file

Additional file 1: Interview guide. Thematic probes used in the interviews. (PDF 58 kb)

## Abbreviation

CMDs: Common mental disorders
