# Supplementary material for: Working in dissonance: experiences of work instability in workers with common mental disorders
Source: BMC Public Health. 2017 May 18;17:472. doi: 10.1186/s12889-017-4388-3 (PMC5437399; doi:10.1186/s12889-017-4388-3)
Supplement: Additional file 1: — Interview guide. Thematic probes used in the interviews. (PDF 58 kb) [file 12889_2017_4388_MOESM1_ESM.pdf]

Interview guide

Thematic probes used in the study *Working in dissonance: experiences of work instability in workers with common mental disorders*

Opening question:

- Can you describe what an ordinary day at work is like for you?

Probes to elaborate on change at work:

- Do you experience any change in how it is for you to work while feeling depressed or anxious? How so?
  - Anything different in how you perform your tasks?
  - Anything different in how your body feels?
  - Anything different in how you interact with others?
  - Were there early signs of change? What came later?

- Can you give an example of a specific situation when the change became clear to you?
- Have you made any adjustments to keep up work? How so?

Probes to elaborate on change outside work:

- Do you experience any change in your life outside work? How so?
- Have you made any changes to your lifestyle, such as activities, social life, food/drinks?

Closing the interview:

- To sum up, what do you think are signs of that the work ability is becoming unstable, based on your own experience?
- Anything else you would like to add to this interview?
